# Lysosome–Iron–Mitochondria Axis in Osteoclasts: Iron as a Central Player

**DOI:** 10.34133/research.0840

**Published:** 2025-08-14

**Authors:** Shengnan Qin, Kathleen Davern, Scott G. Wilson, Kai Chen, Aiguo Li, Jiake Xu

**Affiliations:** ^1^ School of Biomedical Science, The University of Western Australia, Perth, Australia.; ^2^ Harry Perkins Institute of Medical Research, Perth, Australia.; ^3^ Guangzhou Institute of Traumatic Surgery, Guangzhou Red Cross Hospital, Medical College, Jinan University, Guangzhou, China.; ^4^ Department of Endocrinology & Diabetes, Sir Charles Gairdner Hospital, Nedlands, Australia.; ^5^ Department of Twin Research & Genetic Epidemiology, King’s College London, London, UK.; ^6^ Shenzhen Institute of Advanced Technology, Chinese Academic of Sciences, Shenzhen, China.; ^7^ Shenzhen University of Advanced Technology, Shenzhen, China.

## Abstract

Osteoporosis, a widespread skeletal disorder, arises from excessive bone loss, heightening fragility and fracture risk. Osteoclasts, the major type of bone-resorbing cells, are believed to contribute to this loss. Osteoclast bone resorption relies on 2 important organelles: lysosomes for matrix degradation and mitochondria for energy supply. Iron, a critical linker between lysosomes and mitochondria, has emerged as a critical mediator of osteoclast activity. However, the intricate interplay between lysosomes, mitochondria, and iron in osteoclasts and osteoporosis remains poorly understood. This review aims to bridge this knowledge gap by examining the lysosome–iron–mitochondria axis in osteoclasts. Firstly, we summarized the modulatory function of lysosomes in iron metabolism and iron’s involvement in lysosomal biogenesis and function. Next, we conducted a comprehensive analysis on the contribution of iron in mitochondrial function and its implications for osteoclast activity. Subsequently, we highlighted emerging insights into the lysosome–mitochondria crosstalk in iron metabolism. Finally, we delved into the discussion of how dysregulation of this lysosome–iron–mitochondria axis may drive osteoporosis progression and proposed innovative therapeutic strategies targeting this axis to mitigate osteoclast-mediated bone loss.

## Introduction

Osteoporosis is a common skeletal disorder marked by diminished bone mass and structural deterioration, resulting in greater fragility and an elevated risk of fractures. This condition is closely associated with aging, primarily affecting women over 55 and men over 65. In Australia, an estimated 3.4% of the population has osteoporosis or osteopenia, as reported by the Australian Institute of Health and Welfare [1]. In China, the general incidence of osteoporosis is reported to be around 20%, with higher rates in women (23.57%) compared to men (12.22%) [2,3]. However, the actual prevalence is likely underestimated, as osteoporosis is known as a “silent” disease, often presenting no symptoms until fractures occur. Notably, in individuals with severe osteoporosis, particularly of the spine, fractures can occur with little or no trauma, including minor stresses like coughing, sneezing, or bending. Globally, approximately 1 in 3 women and 1 in 5 men over the age of 50 will experience osteoporosis fractures [4].

Osteoclast-mediated bone resorption is a tightly regulated process essential for maintaining skeletal integrity. Hyperactive osteoclasts are recognized as key contributors to excessive bone loss in conditions such as osteoporosis. Osteoclasts possess a unique ability to resorb bone matrix by releasing hydrolytic enzymes and secreting acid into a specialized extracellular compartment known as the ruffled border. This resorptive function is heavily reliant on 2 cellular organelles: lysosomes and mitochondria. Lysosomes create an acidic environment via a vacuolar proton pump (v-ATPase), which is essential for protease production. These acidic components, including protons and proteases, are delivered into the ruffled border to degrade the aged bone matrix [5,6]. Meanwhile, this bone resorption process is highly energy demanding, in which mitochondria serve as the primary energy source. Additionally, mitochondria also provide energy to lysosomes to ensure that they are well functioned in producing and releasing acidic components. For example, v-ATPases use adenosine triphosphate (ATP) hydrolysis energy to pump protons into the lysosome [7]. Except for the energy supply, recent research demonstrated that mitochondrial respiration is also important for maintaining the abundance of cathepsin K (CTSK), one of the most potent proteases released by osteoclasts for bone resorption [8], suggesting that mitochondria can also influence lysosomal function. These findings highlight the interdependence of mitochondria and lysosomes in facilitating effective bone resorption by osteoclasts. However, the potential involvement of a third regulatory component in mediating the crosstalk between lysosomes and mitochondria remains largely unexplored.

A central player linking mitochondria and lysosomes in osteoclast-mediating bone resorption is iron. Lysosomes act as major iron uptake and recycling centers, regulating iron metabolism by controlling its trafficking, storage, and redistribution [9]. Lysosomal acidification is essential for iron uptake, reducing ferric iron (Fe^3+^) to ferrous iron (Fe^2+^), which is then released into the cytoplasm and incorporated into the liable iron pool (LIP) for utilization, storage, or export. Subsequently, mitochondria are the primary sites for iron utilization, facilitating processes, such as oxidative phosphorylation (OXPHOS) and the electron transport chain (ETC) pathway, to generate energy and reactive oxygen species. In the context of bone homeostasis, clinical observations dating back to the early 1900s have established a connection between iron overload and excessive bone loss, underscoring the pivotal role of iron in maintaining bone homeostasis [10]. Osteoclasts, derived from the hematopoietic lineage in the bone marrow [11], reside in an environment that is rich in red blood cells, which require substantial amounts of iron to sustain erythropoiesis [12]. Recent studies have highlighted the importance of iron metabolism in osteoclast activity and bone resorptive function [13–16], indicating that iron is necessary for osteoclastic differentiation and function. Evidence shows that iron chelation can suppress osteoclastogenesis [17–19], while iron overload can promote bone resorption [18,19].

Building on these insights, a deeper understanding of the interactions among lysosomes, mitochondria, and iron, particularly in the context of osteoclasts and osteoporosis, is urgently needed. To date, no comprehensive review has focused on the roles of the lysosome–iron–mitochondria axis in osteoclast function and its implications for osteoporosis. This review aims to address this gap by first examining the evidence supporting the pivotal function of lysosomes in regulating iron homeostasis in osteoclasts, as well as their possible involvement of iron in lysosomal biogenesis and function. Next, we summarize current knowledge on iron utilization in mitochondria and its implications for osteoclast activity. Following this, we explore emerging mechanisms underlying lysosome–mitochondria crosstalk in iron metabolism. Finally, we discuss how dysregulation of the lysosome–iron–mitochondria axis contributes to osteoclast dysfunction and highlight the potential therapeutic strategies targeting this axis for osteoporosis treatment.

## Lysosome–Iron Crosstalk in Osteoclasts

**Fig. 1.**
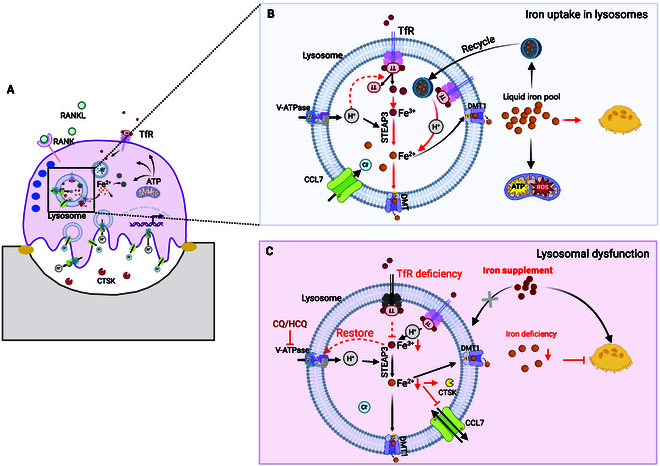
Lysosome–iron crosstalk in osteoclasts. (A) Lysosomes are critical organelles for iron metabolism, which is essential for osteoclast-mediating bone resorption. (B) Lysosomes serve an essential function in intracellular iron uptake, storage, and trafficking. Extracellular ferric iron (Fe^3+^) binds to transferrin (Tf), which interacts with the transferrin receptor (TfR) on the cell membrane and is internalized via endocytosis. Within lysosomes, ferric iron (Fe^3+)^ is reduced to ferrous iron (Fe^2+^) by the ferric reductase STEAP3, a process that depends on the acidic environment maintained by v-ATPase. Fe^2+^ is subsequently exported through divalent metal transporter 1 (DMT1). Due to its cytotoxic potential, Fe^2+^ is either stored in ferritin, which can be recycled and degraded by lysosomes, or utilized by mitochondria to generate energy and reactive oxygen species, both of which are necessary for osteoclast-mediating bone resorption. (C) Lysosomal dysfunction leads to iron deficiency, but iron supplementation cannot restore lysosomal signaling. Disruption of lysosomal acidity by genetical chelation of v-ATPase or v-ATPase inhibitors, such as chloroquine (CQ) or hydroxychloroquine (HCQ), impairs iron uptake in lysosomes, thereby affecting osteoclast function. Meanwhile, TfR deficiency also suppresses osteoclast activity by reducing iron levels and consequently reduces cathepsin production. Notably, iron supplements cannot restore lysosomal pH-related catabolic and signaling function, even though it has been shown to promote osteoclastogenesis. These findings highlight the bidirectional relationship between iron metabolism and lysosomal function in osteoclasts, underscoring their critical interplay in osteoclast-mediating bone resorption. [Created in BioRender. Qin, I. (2025) https://BioRender.com/x08u220.]

Lysosomes are indispensable organelles for osteoclast-mediated bone resorption, providing an acidic environment and specialized enzymes that degrade the bone matrix. This acidic environment is essential for iron homeostasis, facilitating iron uptake, trafficking, and recycling. Disruption of lysosomal acidification impairs iron homeostasis in osteoclasts, thereby affecting bone resorption, which is underscored by genetic mutations in lysosomal-related genes, which cause osteoclast-rich osteopetrosis [20], a disorder where there are normal or increased numbers of osteoclasts, but these cells are unable to resorb bone properly. Conversely, iron may play a regulatory role in lysosomal biogenesis and function, potentially influencing osteoclast activity through a complex feedback mechanism. The bidirectional relationship between iron metabolism and lysosomal function in osteoclasts underscores their critical interplay in osteoclast-mediating bone resorption (Fig. 1).

### Lysosomes as essential organelles in osteoclast bone resorption

Osteoclasts are the primary cells responsible for bone resorption, and their activity relies heavily on lysosomal degradation. Lysosomes are acidic, membrane-bound organelles enriched with hydrolytic enzymes, functioning as central hubs for the degradation and recycling of intra- or extracellular materials [9]. In osteoclasts, lysosomes play a critical role in bone resorption by creating acidic environments and secreting enzymes that break down the bone matrix (Fig. 1A) [5,21]. To create an acidic environment, lysosomes use membrane proteins like the v-type proton ATPase 116 kDa subunit a 2 (ATP6V0A2) and chloride voltage-gated channel 7 (CLCN7) to secrete H^+^ and CI^−^ into ruffled border [22]. Meanwhile, lysosomes secrete soluble enzymes like cathepsins B, K, and L, which are active at low pH and degrade the organic matrix, particularly type I collagen [22]. CTSK is the most abundant lysosomal cysteine protease in osteoclasts. It is responsible for degrading organic bone matrix, such as collagens, lamin, tenascin, and osteonectin [23]. Notably, although lysosomes have a limited role in hydroxyapatite dissolution, lysosomes contribute to the acidification necessary for the dissolution of hydroxyapatite. During bone resorption, lysosomes fuse with the osteoclast plasma membrane at the ruffled border, releasing their contents, including H^+^ and CI^−^, to create the acidic environment necessary for hydroxyapatite dissolution. Therefore, lysosomes are essential for both degrading the organic matrix and acidifying environment necessary for hydroxyapatite dissolution in the osteoclast-mediated bone resorption.

### Acidic lysosomes are master regulators of iron homeostasis in osteoclasts

Lysosomal acidification is necessary for maintaining iron homeostasis within the cell, ensuring that iron is available for essential cellular processes while preventing its accumulation in toxic forms [9,24]. Lysosomes are the first organelles to process extracellular iron imported via endocytosis, and lysosomal acidification is necessary for iron uptake, storage, and trafficking [9]. The precise mechanism by which lysosomal acidification influences iron uptake in osteoclasts remains unclear. However, insights from studies in other cell types provide a clearer understanding of lysosomal iron processing (Fig. 1B). It is known that extracellular Fe^3+^ is first bound by transferrin (Tf), which facilitates its cellular uptake through interaction with the transferrin receptor (TfR) and subsequent endocytosis. Then, the TfR–Tf–Fe complex may be disassembled by an acidic environment to release irons. After being imported into lysosomes, ferric iron (Fe^3+^) is reduced to its ferrous form (Fe^2+^) by the ferric reductase 6-transmembrane epithelial antigen of prostate 3 (metalloreductase STEAP3), which also requires an acidic environment maintained by lysosomal proton pumps, particularly the v-ATPase [9]. However, intracellular free Fe^2+^ can be highly toxic and needs to be exported through specialized transporters, such as divalent metal transporter 1 (DMT1) [25], for storage by ferritin or utilization by mitochondria. It is worth noting that DMT1 increases during osteoclastogenesis, and overexpression of DMT1 in turn promoted osteoclast differentiation and caused iron accumulation in osteoporosis mice [26], indicating that lysosome-mediated iron transport plays a crucial role in regulating osteoclast activity. To maintain iron homeostasis, lysosomes can also recycle iron by breaking down iron-containing molecules, particularly from ferritin or aged organelles such as mitochondria [24,27]. In addition to their established functions in degradation and recycling, the lysosome has been recognized as a sensor hub of iron-associated metabolic signaling. Nevertheless, the intracellular mechanisms underlying the interaction between lysosomal function and iron metabolism in controlling essential cellular activities are still not fully understood [9].

Dysfunction of lysosomal acidification can markedly affect iron uptake by disrupting the transformation from Fe^3+^ to Fe^2+^ as well as the iron release from the TfR–Tf–Fe complex and ferritin (Fig. 1C). As mentioned above, the vacuolar-ATPase complex is responsible for H^+^ release and creates an acidic environment in lysosomes. It plays a crucial role in regulating cellular iron levels and disrupting the v-ATPase complex, or its assembly factors lead to iron depletion [28]. Genetic ablation of v-ATPase leads to intracellular iron depletion, which supports the essential function of lysosomal acidification in iron metabolism [28]. Additionally, pharmacological interventions targeting lysosomal pH, such as chloroquine (CQ) and hydroxychloroquine (HCQ), have demonstrated potential in disrupting iron homeostasis and osteoclast activity. These lysosomotropic drugs increase lysosomal pH, thereby preventing the release of iron from ferrated Tf and interfering with iron trafficking to the cytoplasm [29,30]. Both drugs suppressed osteoclast differentiation and bone resorption [31], suggesting that lysosomal pH modulation may be the reason for iron deficiency, ultimately resulting in bone resorption dysfunction. However, additional research is required to elucidate whether there is a direct regulation between osteoclast dysfunction and iron imbalance. Except for the transformation of iron, lysosomal acidification is also vital for the release of Fe^3+^ from the TfR–Tf–Fe complex and the recycling of Fe^2+^ from ferritin [32–34]. A deficiency in TfR has also been shown to reduce iron uptake notably [13,35] and impair osteoclast formation and resorptive function [13] (Fig. 1C). Lysosomal acidification may also play a crucial role in the processing and recycling of TfR.

### Does iron reversely regulate lysosomal biogenesis and function within osteoclasts?

Although it is reported that iron supplementation cannot restore lysosomal pH-related signaling functions, iron indeed influences lysosomal biogenesis and function. Both iron deficiency and iron overload could disrupt lysosomal biogenesis and function [34,36–38].

Iron deficiency can lead to decreased autophagy and impaired lysosomal biogenesis due to the reduced availability of iron for the posttranslational maturation of lysosomal membrane proteins [36]. In terms of osteoclasts, direct studies on the effects of iron on lysosomal biogenesis are lacking. Still, evidence suggests that iron levels may influence lysosomal degradation function due to the facts that iron deficiency impaired osteoclast-mediating bone resorption [13,25,35]. Similarly, iron chelation inhibited osteoclastic bone resorption and protected against bone loss following estrogen deficiency resulting from ovariectomy [39]. In addition, TfR1-mediated iron uptake plays a vital role in regulating bone mass in mice, especially in osteoclasts [13]. Specifically, TfR1 facilitates iron uptake, which is governed by lysosomal acidification in osteoclasts, ultimately influencing bone resorption and bone mass. Deletion of TfR1 in osteoclasts leads to increased trabecular bone mass [13], indicating that TfR1-mediated iron uptake is important for bone resorption, during which lysosomes are vital, as mentioned above.

Reversely, iron overload can also impact the lysosomal degradation function. It can lead to impaired lysosomal acidification and potentially increased lysosomal numbers [37]. This can result in the accumulation of iron within the lysosomes, leading to an increase in lysosomal pH [37]. In some cases, iron overload can lead to more lysosomal biogenesis, potentially as a compensatory mechanism to handle the increased iron burden [37]. For osteoclasts, iron overload can induce osteoporosis by promoting their differentiation and bone resorption [18], demonstrating that iron overload promotes more bone matrix removal, which needs more proton and proteinase from lysosomes into ruffled border. Additionally, prazosin, a drug that increases iron levels in bone tissue, also affected the expression of CTSK and CLCN7, 2 predominantly osteoclastic lysosomal proteases, in bone tissue [40], indicating that the change in iron levels influenced the expression of lysosomal proteins in osteoclasts.

Iron regulatory protein 2 (IRP2), a key regulator of intracellular iron homeostasis [41], is required for lysosomal acidification and biogenesis [42]. IRP2 deficiency reduced the nuclear translocation of transcription factor EB (TFEB), a master regulator of lysosomal biogenesis, thereby down-regulating lysosome-related genes involved in iron metabolism and lysosomal components [42]. In addition, lysosomes are implicated in ferroptosis, a form of cell death driven by lipid-based reactive oxygen species, where iron overload can trigger this process [43]. Collectively, these findings demonstrate the importance of iron in lysosomal biogenesis and function within osteoclasts; however, further research and the underlying mechanism warrant investigation.

## Iron Utilization in Mitochondria in Osteoclasts

**Fig. 2.**
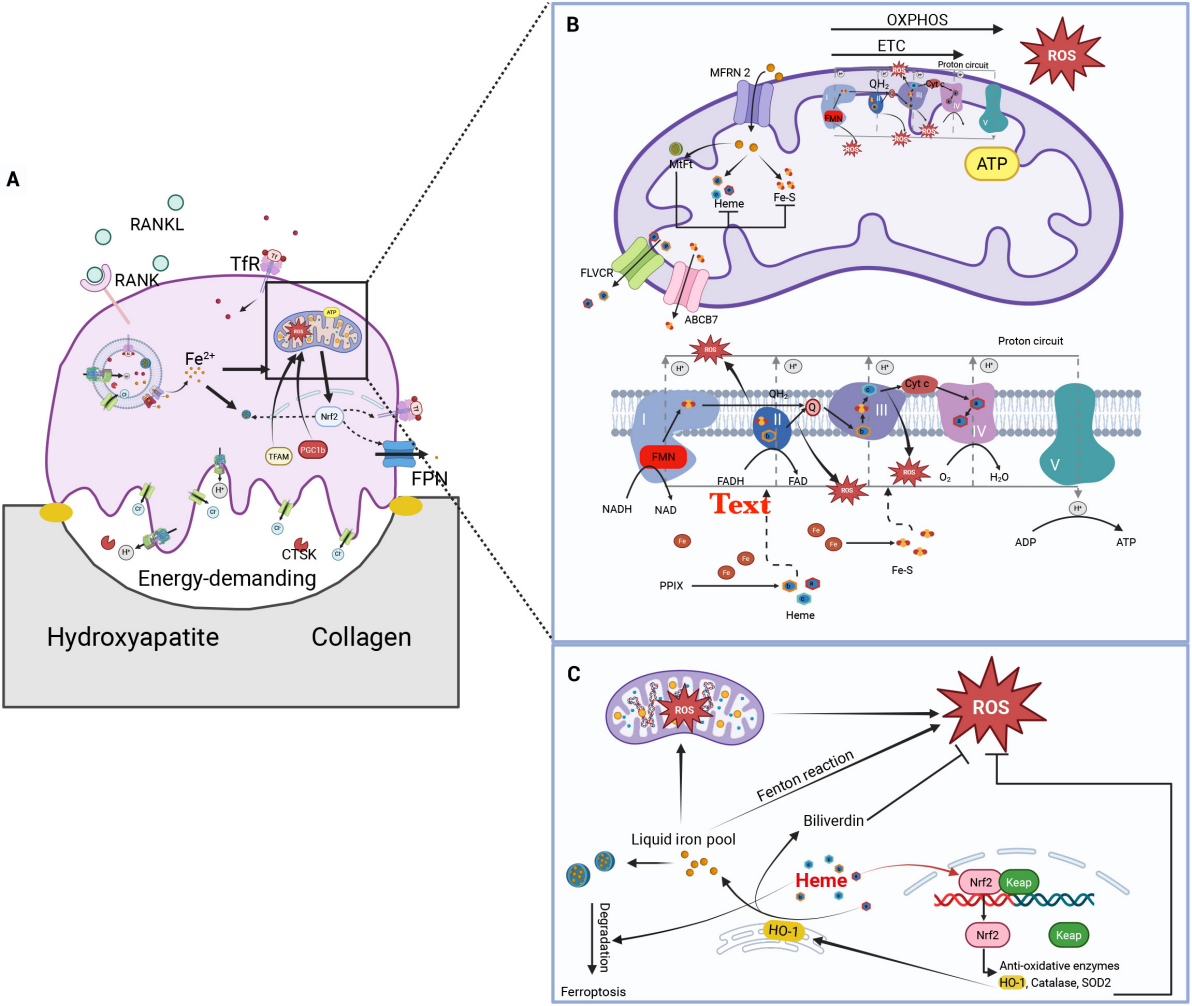
Iron–mitochondria crosstalk within osteoclasts. (A) Osteoclast-mediating bone resorption is a highly energy-demanding process requiring substantial ATP. (B) Mitochondria require iron for energy production and ROS generation through oxidative phosphorylation (OXPHOS). Iron is imported via mitofusin-2 (MFN2) and is subsequently utilized for the synthesis of iron–sulfur (Fe–S) clusters and heme, both of which are cofactors for mitochondrial respiratory complexes I to IV. Lastly, heme can be exported from mitochondria via FLVCR. (C) Iron contributes to intracellular ROS production, which is crucial for osteoclast-mediated bone resorption through 2 mechanisms: Fenton reaction and mitochondrial ROS generation. Excessive intracellular ROS is toxic for cells, and heme may play a critical role in balancing the ROS within osteoclasts: (1) it can be degraded by heme oxygenase-1 (HO-1), releasing Fe^2+^ and biliverdin, which is an antioxidant component; (2) it may participate in ferritin degradation, potentially resulting in ferroptosis; and (3) it may activate Nrf2 signaling, which in turn induces HO-1 expression. Created in BioRender. Qin, I. (2025) https://BioRender.com/e87p651.

Osteoclast-mediated bone resorption is a highly energy-demanding process, as bones are composed of strong, mineralized tissue that provides support and protection (Fig. 2A). They are composed of collagen and mineral salts, which contribute to their rigidity [44]. Osteoclasts need substantial energy to dissolve bone minerals, degrade collagen, and maintain their high motility. Several steps are involved in bone resorption, including the active extrusion of protons to dissolve hydroxyapatite mineral, the secretion of CTSK to degrade collagen, and the maintenance of a ruffled border for efficient resorption.

Osteoclasts contain numerous mitochondria and rely heavily on them for energy production and reactive oxidative species (ROS) generation (Fig. 2B). Iron is essential for mitochondria function, particularly in the synthesis of heme and iron–sulfur (Fe–S) clusters (Fig. 2C). Iron deficiency disrupts mitochondrial OXPHOS and the ETC, impairing the energy supply for osteoclast-mediated bone resorption. Iron is also necessary for mitochondrial ROS generation, which is the primary source of intracellular ROS and serves a crucial role in osteoclast activation and function. In addition to energy metabolism and ROS, the links between mitochondria and iron within osteoclasts are becoming clearer. For example, the hidden link between heme-iron-HO1 and Nrf2 signaling is being increasingly revealed in regulation of oxidative stress and iron homeostasis within osteoclasts.

### Mitochondrial requirements for iron for energy production through OXPHOS

Mitochondrial respiration and glycolysis represent the 2 primary metabolic pathways that generate ATP from nutrients, fueling essential cellular processes [45]. Although it is reported that metabolic reprogramming in osteoclasts relies on glycolysis more, osteoclast function maintenance requires OXPHOS [46]. It is believed that glycolysis, occurring in the cytoplasm, breaks down glucose into pyruvate, producing a small amount of ATP. Pyruvate then enters the mitochondria, where it is further processed via the citric acid cycle and OXPHOS, yielding a much larger amount of ATP [47]. Because osteoclast-mediated bone resorption is a highly energy-demanding process, it is logical that osteoclasts rely heavily on their abundant mitochondria for ATP generation [48].

Iron plays a pivotal role in energy metabolism in mitochondria via OXPHOS [9]. Iron deficiency can directly damage OXPHOS [49], producing less energy [50]. Dysfunction of mitochondria due to impaired OXPHOS can result in osteoclast dysfunction. For example, *Ndufs4* knockout osteoclasts, which lack a subunit of complex I in the ETC, exhibited impaired osteoclast formation and bone resorptive activity [51]. Additionally, PGC1β, a key factor in iron uptake [19], can impair OXPHOS. Specifically, PGC1β deficiency leads to reduced OXPHOS gene expression, decreased mitochondrial size, and impaired mitochondrial oxygen consumption and ATP synthesis [52–54]. Meanwhile, PGC1β-deficient osteoclasts also exhibit an iron imbalance and impaired bone resorption function [39,55].

Although the molecular mechanisms governing iron delivery and utilization in mitochondria remain incompletely understood, iron in mitochondria undergoes several steps as outlined below (Fig. 2B). Mitoferrin 2 (MFRN2; also known as *SLC25A28*) has been identified as the principal mitochondrial iron importer in non-erythroid tissues [12] and positively contributes to RANKL-activated osteoclast formation [56]. After iron is transported into mitochondria, it is involved in the biosynthesis of heme and Fe–S clusters, which serve as cofactors in OXPHOS [12].

Mitochondrial OXPHOS complexes are a group of 5 protein complexes embedded in the inner mitochondrial membrane that work together to generate ATP, the primary energy source for the cell. Heme is found in complexes II (succinate dehydrogenase), III (ubiquinol-cytochrome reductase), and IV (cytochrome c oxidase), facilitating redox reactions and electron transfer [57,58]. Excessive heme is exported by feline leukemia virus subgroup C receptor 1b (FLVCR1b), the main transporter, which is responsible for exporting heme from mitochondria, and subsequently, heme in the cytosol is exported by FLVCR1a [59,60]. This heme export is crucial for various cellular processes, including protecting cells from heme toxicity, recycling iron from heme degradation, and regulating heme synthesis and degradation [60]. Heme synthesis and degradation pathways also influence osteoclastogenesis, and their degradation can contribute to bone loss [61,62]. Fe–S clusters are found in complexes I [reduced form of nicotinamide adenine dinucleotide (oxidized form) (NADH) dehydrogenase], II, and III. Fe–S clusters act as electron carriers, facilitating the transfer of electrons along the ETC [63]. The export of Fe–S clusters from mitochondria is mediated by the transporter ABCB7 [64]. However, the precise role of the Fe–S cluster in osteoclast formation and bone resorption remains under-explored to date. Additionally, iron is also a constituent of cytochromes, proteins that contain heme and use electron transfer to oxidize and reduce iron [65].

Meanwhile, excessive mitochondrial iron retention induces oxidative stress, leading to mitochondrial damage [12,66–68]. To avoid this damage, mitochondria can utilize their own ferritin, known as mitochondrial ferritin (MtFt), which structurally and functionally resembles cytosolic ferritin. MtFt is predominantly composed of H-chains, suggesting that its primary role is to sequester ferrous iron (Fe^2+^) within the mitochondria, thereby avoiding superoxide production and preventing Fenton reactions [69]. MtFt overexpression induces a dose-dependent cytosolic iron deficiency, markedly enhances cellular iron uptake from Tf, and restricts the synthesis of heme and Fe–S clusters [70]. However, MtFt expression is limited to specific cell types [69], and its presence in osteoclasts has not yet been reported, warranting further investigation.

### Iron is essential for mitochondrial ROS generation

Reactive oxygen species (ROS) are fundamental to osteoclast activities [71–75], stimulating differentiation and facilitating bone resorption. There are 2 ways in which iron is involved in ROS production in cells (Fig. 1C). Iron is essential in mitochondrial ROS production, including that in the ETC and OXPHOS [76]. Iron can also produce ROS by catalyzing the Fenton reaction, a redox reaction that produces hydroxyl radicals (OH) from the combination of Fe^2+^ and hydrogen peroxide (H_2_O_2_) [77].

During OXPHOS in mitochondria, iron, as a key component of the ETC, facilitates the transfer of electrons, generating ROS across the inner mitochondrial membrane. In the ferrous state (Fe^2+^), iron can donate electrons, while in the ferric state (Fe^3+^), it can accept electrons. This ability is fundamental for mediating redox reactions and generating ROS within the ETC complexes I, II, and III of the mitochondria (Fig. 2B). While all complexes are involved in electron transfer and proton pumping, iron’s redox activity, especially in the Fe–S clusters, can lead to the production of superoxide and other ROS [78–80]. Specifically, complex I facilitates electron transfer from NADH to ubiquinone, also known as coenzyme Q (CoQ), generating ROS as a byproduct. Complex II oxidizes succinate to fumarate and transfers electrons to CoQ, producing ROS but much less than complex I. Complex III transfers electrons from CoQ to cytochrome c and can be a source of ROS.

Elucidating the effects of ROS during osteoclastogenesis has proven to be an efficient solution for osteoporosis treatment in murine models [81]. Mitochondria represent a key site of ROS production, which is a byproduct of OXPHOS as mentioned above (Fig. 2B). Increased mitochondrial ROS levels correlate with osteoclastogenesis [82]. While the specific contributions of mitochondrial ROS to osteoclast biology are not fully elucidated, mitochondria are estimated to generate approximately 90% of intracellular ROS [83]. In parallel with strategies targeting intracellular ROS to suppress osteoclast activity, selective reduction of mitochondrial ROS has also been shown to inhibit osteoclast differentiation and formation, thereby alleviating bone loss in mice. For example, metallothionein 3 (MT3), a metal-binding protein involved in cellular metal homeostasis, regulates mitochondrial ROS levels. MT3 depletion in osteoclast precursors reduces mitochondrial ROS, consequently inhibiting osteoclast differentiation [82].

Despite the well-established role of iron in promoting ROS production, as mentioned above, it may also play a dual game in regulating intracellular oxidative stress, potentially protecting cells from excessive ROS exposure.

### The hidden link between Nrf2 and heme-HO-1 by iron

Heme, a primary functional form of iron, is synthesized in the mitochondria by ferrochelatase, which inserts Fe^2+^ into protoporphyrin IX [84]. Heme metabolism, particularly through the activity of heme oxygenase-1 (HO-1), plays a crucial role in osteoclast differentiation and bone resorption [61,62,85]. Once released from mitochondria, free heme is cytotoxic and must be rapidly degraded by HO-1 into carbon monoxide, biliverdin, and free iron [62,86–88]. Interestingly, during osteoclastogenesis, HO-1 decreases, suggesting a change in the demand for heme degradation. Meanwhile, activation of HO-1 has been shown to inhibit osteoclast differentiation [89]. Among the products of heme degradation, biliverdin is recognized to account for the anti-oxidative and anti-inflammatory properties of HO-1. In contrast, the released free iron contributes to ROS production via the Fenton reaction or mitochondrial OXPHOS pathways. Thus, the products of heme degradation exhibit opposing effects on ROS levels—an aspect that has been largely overlooked—suggesting that the HO-1–heme–iron axis may act as a vital mediator in maintaining intracellular ROS balance.

Several lines of seemingly conflicting evidence support this hypothesis, revealing the multifaceted role of heme in osteoclast biology. For instance, a recent study has shown that heme synthesis and metabolism pathways are activated during osteoclastogenesis [61]. Inhibiting heme synthesis can effectively hamper RANKL-induced osteoclast formation and mitigate bone loss caused by ovariectomy (OVX) [61], indicating that the free iron released from heme degradation may play a more important role in osteoclasts and might well abolish the anti-oxidative effects of biliverdin. Conversely, heme has also been reported to activate the Nrf2 signaling pathway [90], which in turn induces a suite of antioxidant enzymes, including HO-1, that inhibit osteoclast differentiation and function [91,92]. Additionally, iron deficiency can also lead to heme deficiency, further impairing mitochondrial energy production and increasing oxidative damage [84], ultimately affecting osteoclast activity.

These findings suggest a complex interplay between heme metabolism, iron homeostasis, and the Nrf2–HO-1 axis. Specifically, Nrf2 may act as a key regulatory node in this network, coordinating the response to intracellular iron levels and oxidative stress (Fig. 2C). Emerging evidence supports Nrf2’s role in iron metabolism across various cell types, including osteoclasts [91–95]. Nrf2 activation decreases iron availability by regulating the expression of genes involved in iron uptake, storage, and export [93,95,96]. Meanwhile, Nrf2 activators, such as bitopertin, dimethyl fumarate, and sulforaphane, show promise as a therapeutic strategy for osteoporosis, particularly in suppressing osteoclast differentiation and bone resorption [91,94,97–99]. By activating Nrf2, intracellular iron levels can be reduced in osteoclasts, thereby inhibiting their activity and preventing bone loss. Normally, Nrf2 is inactivated in osteoclastogenesis, but under iron overload conditions, Nrf2 activation appears to be essential for osteoclast differentiation [16], suggesting a context-dependent or bidirectional relationship between iron levels and Nrf2 signaling.

Taken together, these observations reveal a potentially critical but underexplored link between Nrf2, heme, HO-1, and iron in the underlying mechanism of Nrf2 activators in osteoclasts (Fig. 2C). Nrf2 activators have been shown to inhibit osteoclasts, and the underlying mechanism might be that activation of Nrf2 leads to increased HO-1, which in turn degrades heme and releases free iron, creating a feedback loop that regulates intracellular iron and ROS levels. Under iron overload, the reason why Nrf2 activation is necessary for osteoclast differentiation [16] may be that iron overload induces increased heme synthesis, which activates Nrf2 in response to heme-stress and processes the excessive iron, thereby increasing ROS levels. Nevertheless, the precise role of the iron–heme–Nrf2–HO-1 axis in osteoclast function remains unclear. Although Nrf2 activators have demonstrated protective effects against osteoclast activity by modulating iron metabolism, whether iron exerts a link between Nrf2 and heme-HO-1 remains an open question.

## Mechanisms of the Lysosome–Mitochondria Crosstalk in Iron Metabolism

Genetic and molecular studies highlight the intricate interplay between mitochondria and lysosomal compartments, revealing that their functions are closely linked and mutually influential. Lysosomal dysfunction directly induces mitochondrial dysfunction, and some microphthalmia-associated transcription factor (MiT) family members, such as MITF, TFE3, and TFEB, also regulate lysosomal biogenesis. Understanding the role of iron in this organelle interconnection offers promising therapeutic targets for addressing osteoclast-induced bone loss.

### Iron trafficking between lysosomes and mitochondria

**Fig. 3.**
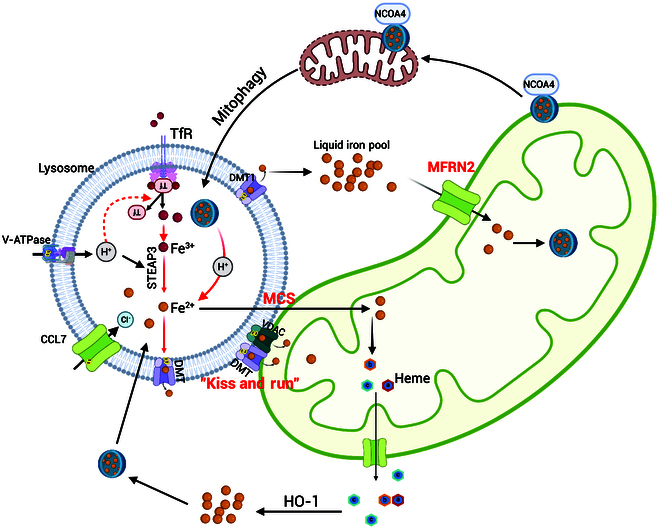
Iron trafficking between lysosomes and mitochondria. Iron plays a critical role in lysosome–mitochondria cross-talk. While the mechanisms governing iron trafficking from lysosomes to mitochondria remain incompletely defined, 2 primary pathways have been identified: mitoferrin-mediated import and “kiss and run”. Additionally, membrane contact sites (MCSs) have been recently identified as potential mediators of iron transfer between these organelles. Conversely, mitochondria can transport iron back to lysosomes for recycling through NCOA4-mediated mitophagy and the release of heme. Created in BioRender. Qin, I. (2025) https://BioRender.com/s52g771.

Iron trafficking from lysosomes to mitochondria remains incompletely characterized, but 2 primary mechanisms have been identified (Fig. 3).

Mitoferrin-mediated import [100]: Upon lysosomal release of Fe^2+^, mitoferritin (MFRN2 in non-erythroid tissues) acts as the primary mitochondrial iron importer and is located in the inner mitochondrial membrane. MFRN2 is stabilized by ATP-binding cassette sub-family B member 10 (ABCB10) [10,18], ensuring efficient iron transport into the mitochondrial matrix for essential processes such as heme synthesis and Fe–S cluster formation.

“Kiss and run” [101–103]: In this mechanism, lysosomes transfer iron to mitochondria via direct contact with Tf-bound endosomes [75–77]. This process involves key proteins, such as DMT1, which localizes to both the lysosomal and outer mitochondrial membranes, facilitating iron transfer. The voltage-dependent anion-selective channel (VDAC1), which colocalizes with DMT1 [104], supports the docking process, thereby further enabling efficient iron trafficking. Additionally, membrane contact sites (MCSs), recently identified as potential mediators of iron transfer [105], play a role in lysosomal iron storage in facilitating mitochondrial iron uptake.

Interestingly, mitochondria can transport iron back to lysosomes for recycling. Lysosomes are the prominent organelles responsible for recycling iron from aged mitochondria through mitophagy [9], especially in nucleated cells [24]. MtFt functions as a mediator of mitophagy triggered by low-iron conditions through lysosomal degradation of mitochondria [106]. During iron starvation, mitochondrial damage leads to the accumulation of MtFt on the outer mitochondrial membrane, where its interaction with the cargo receptor NCOA4 promotes the tethering of mitochondria to the expanding autophagosome, ultimately enhancing mitophagy [107]. In addition, heme released from mitochondria is degraded by HO-1 to free iron, which is stored in ferritin and reused by lysosomes. Mitochondria may also transport cargoes to lysosomes via mitochondrial-derived vesicles (MDVs) [108], and these vesicles may also transport iron to lysosomes, although this requires further investigation.

### Cellular signaling pathways that are involved in lysosome–iron–mitochondria crosstalk

The interplay between iron, lysosomes, and mitochondria is mediated by several signaling cascades involving calcium (Ca^2+^), mammalian target of rapamycin (mTOR) signaling, hypoxia-inducible factor (HIF) signaling, and adenosine monophosphate-activated protein kinase (AMPK) signaling.

**Fig. 4.**
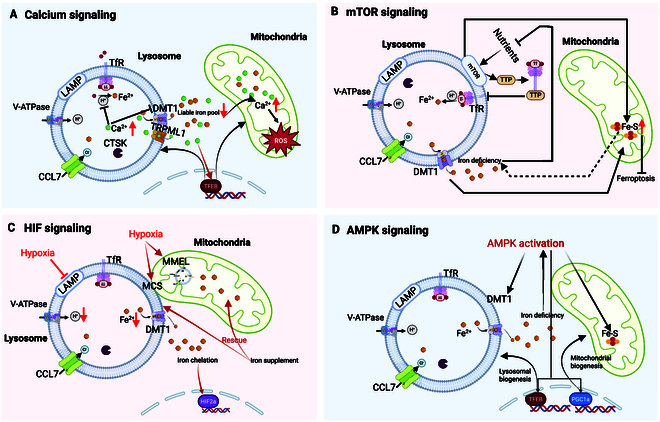
Cellular signaling pathways that are involved in lysosome–iron–mitochondria crosstalk. (A) Calcium signaling plays a key role in regulating iron homeostasis by influencing lysosomal and mitochondrial functions. Elevated calcium levels can disrupt iron transport and organelle function by competing with iron for receptors, altering receptor localization, and affecting lysosomal acidification. The TRPML1 channel mediates lysosomal calcium release, activating TFEB to enhance lysosomal and mitochondrial function, thereby helping counteract iron overload-induced cytotoxicity. (B) mTORC1 signaling regulates iron homeostasis and osteoclast function by coordinating lysosomal and mitochondrial activity. Iron levels influence mTORC1 activation at lysosomes, while mTORC1, in turn, modulates TfR-mediating iron uptake by TTP. mTORC1 can also increase Fe–S synthesis, which inhibits ferroptosis. (C) HIF signaling links hypoxia with iron metabolism, lysosomal function, and mitochondrial dynamics. Hypoxia impairs lysosomes, alters iron regulation, and induces processes such as MMEL and MSD, thereby increasing ROS. Iron depletion activates HIF, while iron supplementation can restore mitochondrial function under hypoxic stress. (D) AMPK signaling is activated under iron deficiency and regulates iron metabolism by affecting iron transport, storage, and utilization. It influences key pathways, including DMT1 expression, HIF-1α, IRPs, and Nrf2 signaling, and promotes lysosomal and mitochondrial biogenesis through TFEB and PGC1α. [Created in BioRender. Qin, I. (2025) https://BioRender.com/smhyfjs.]

Calcium signaling: The activation of calcium signaling plays a crucial role in iron homeostasis, particularly in lysosomes and mitochondria (Fig. 4A). Elevated lysosomal or mitochondrial calcium levels disrupt iron homeostasis and contribute to these organelle dysfunctions [109]. Calcium signaling affects iron uptake by binding to and competing with iron for the same receptor sites [110]. For example, calcium can cause the internalization of DMT1 receptors (localized on lysosomes and mitochondria), limiting the transfer of iron into enterocytes [111]. In addition, calcium has been shown to influence lysosomal acidification [112], which could impact iron uptake and, consequently, induce mitochondrial dysfunction, oxidative stress, and, ultimately, osteoclast dysfunction. Transient receptor potential mucolipin 1 (TRPML1) is the best-known calcium channel in the lysosome [113]. Through TRPML1, calcium can be released from lysosomes, activating TFEB, a master regulator of lysosomal biogenesis and mitochondrial metabolism [114,115]. Increasing TFEB levels, in turn, reverts the iron overload-associated cytotoxicity, dependent on a TRPML1-mediated increase in cytosolic calcium levels [116].

mTOR signaling: Lysosomes and mitochondria work closely together, and this coordination is largely controlled by mTOR signaling, a central hub that regulates both iron homeostasis [117–119] and osteoclast biology [120–123] (Fig. 4B). mTORC1, the active form located on lysosomal membranes, is essential for osteoclast activity and mitochondrial function. Its activation is closely linked to iron availability; iron deficiency or chelation suppresses mTORC1 activity, affecting lysosome-sensing nutrients [124], whereas iron overload impairs mTORC1 signaling and blocks autophagic flux [125]. mTORC1 not only responds to iron but also regulates its homeostasis [117]. It promotes the synthesis of Fe–S clusters, thereby suppressing the iron regulatory protein/iron-responsive element (IRP/IRE) pathways, reducing intracellular free iron, and preventing ferroptosis [126]. mTORC1 also affects iron turnover through tristetraprolin (TTP) [117] and modulates autophagy, which governs lysosomal iron release and ferritin degradation via ferritinophagy [127]. Inhibition of mTOR leads to increased lysosomal iron release, potentially contributing to ferroptosis. Overall, mTORC1 acts as critical signaling node connecting iron regulation, lysosomal activity, and mitochondrial function, highlighting its central role in the lysosome–iron–mitochondria crosstalk.

HIF signaling: Hypoxia has a complex interplay between iron homeostasis, lysosomal function, and mitochondrial dynamics under low oxygen conditions (Fig. 4C). Hypoxia alters iron homeostasis by modulating IRPs [128–130] and impairs lysosomal function by increasing lysosomal pH and inducing structural changes [131,132], which can disrupt iron uptake and storage. In response to hypoxia, mitochondria undergo remodeling and form enhanced contacts (MCS) with lysosomes. Specifically, hypoxia can trigger the engulfment of lysosomes by mitochondria, a process called megamitochondria engulfing lysosomes (MMELs) [133], which may lead to mitochondrial self-digestion (MSD) and elevated ROS production [133,134]. Moreover, iron depletion, often accompanying hypoxia, further activates HIF signaling due to impaired lysosomal and mitochondrial function [12]. Conversely, iron supplementation under hypoxic stress can restore mitochondrial integrity and reduce cellular damage [135]. Together, these findings reveal a tightly integrated response involving HIF signaling, iron availability, and lysosomal–mitochondrial interactions during hypoxic stress, highlighting the role of HIF as a central regulator in the lysosome–iron–mitochondria axis.

AMPK signaling: AMPK is a key energy sensor that is activated under conditions of low energy, including iron deficiency [136] (Fig. 4D). Once activated, AMPK initiates a series of downstream responses that influence iron uptake, storage, and utilization. One major effect is the regulation of DMT1, a transporter critical for iron release from lysosomes to cytosol [137]. Reduced DMT1 expression, in turn, activates IRPs, further modulating intracellular iron levels [137]. AMPK also indirectly affects iron metabolism through HIF-1α, as reduced AMPK activity can promote HIF signaling, particularly under hypoxic or iron-deficient conditions. Moreover, AMPK influences mitochondrial iron handling by regulating ISCU, a key Fe–S cluster assembly enzyme, and ALAS2, involved in heme biosynthesis [138]. These effects collectively shape mitochondrial composition and function. In addition, AMPK activates Nrf2 signaling [139], a master regulator of antioxidant defense and iron metabolism. It also promotes TFEB-dependent lysosomal biogenesis and PGC1α-mediated mitochondrial biogenesis, enhancing both lysosomal function and mitochondrial capacity under energy or iron stress [140].

Further studies are necessary to elucidate the intricate regulatory networks that link mitochondrial iron metabolism, ROS generation, and osteoclast function, providing novel insights for therapeutic strategies in osteoporosis.

## Lysosome–Iron–Mitochondria Axis in Osteoclasts and Osteoporosis

### The lysosome–iron–mitochondria axis in osteoclasts and osteoporosis

The lysosome–iron–mitochondria axis refers to the integrated cellular network that governs the trafficking, utilization, and recycling of iron between lysosomes and mitochondria. Functionally, this axis is crucial for maintaining mitochondrial energy metabolism and redox balance, and it is especially relevant in osteoclasts due to their high metabolic and iron-handling demands. Key molecular mediators of this axis include transferrin receptor 1 (TfR1) for iron uptake, DMT1 for lysosomal iron export, MFRN2 for mitochondrial iron import, VDAC3 for inter-organelle iron transfer, and TFEB for transcriptional coordination of lysosomal and mitochondrial biogenesis. In addition, Rab7 also MCSs (Fig. 3), possibly affecting the iron transfer from lysosomes to mitochondria.

Emerging evidence indicates that disruption of any component of this axis (e.g., via DMT1, TfR1, MFRN2, or TFEB perturbation) in osteoclasts leads to impaired osteoclast differentiation and/or bone resorption, underscoring the functional specificity of this axis for osteoclast activity. TfR1 is a protein that mediates cellular iron uptake through the endocytosis of iron-loaded Tf. During osteoclast differentiation, TfR1 expression increases and disruption of TfR1 expression in osteoclasts results in decreased bone resorption [13]. DMT1 is a key transporter that is located in lysosomes and also increases during osteoclastogenesis [26,141] and osteoporosis in mice [142]. DMT1 may influence osteoclast activity as evidenced by the finding that overexpression of DMT1 in osteoclasts leads to enhanced differentiation [26], while reducing its expression in diabetic osteoporotic mice prevents bone loss [142]. MFRN2 is a protein in the inner mitochondrial membrane responsible for transporting iron into mitochondria. The tethering function of MFRN2 controls osteoclast differentiation by modulating the calcium signaling pathway and the activation of NFATc1 [143]. Meanwhile, depleting MFRN2 in osteoclasts leads to increased bone mass due to reduced bone resorption [143]. VDAC, a voltage-dependent anion channel, is found in both the mitochondria and cell membrane of human osteoclasts [144]. Anti-VDAC antibody shows inhibitory effects on osteoclastogenesis via NFATc1 [144]. TFEB plays a crucial role in osteoclast function, particularly in lysosomal biogenesis and bone resorption [145], as it is the master regulator of lysosomal and mitochondrial biogenesis as mentioned above. Additionally, osteoclasts derived from lysosomal storage disease models or with mitochondrial dysfunction (e.g., PGC1β or Ndufs4 deficiency) exhibit defective resorption, while macrophage counterparts remain relatively unaffected [51,55], supporting osteoclast-specific sensitivity.

These mechanistic insights into the lysosome–iron–mitochondria axis and its key molecular players highlight its fundamental role in osteoclast differentiation and resorptive function. Given the axis’s sensitivity to iron availability and organelle coordination, its dysregulation may underlie pathological conditions of excessive bone loss. Indeed, iron overload has long been linked with compromised bone integrity. Elevated body iron levels are recognized as an independent risk factor for accelerated bone loss in several conditions, including hemochromatosis [146–148], sickle cell disease [149], and postmenopausal women [150,151]. Experimental animal models have consistently demonstrated that iron overload induces bone weakening, with the osteoporotic phenotype in iron-overloaded mice being associated with an increased number of osteoclasts in bone tissue [152,153]. Hepcidin deficiency, which leads to iron overload, consistently results in elevated levels of type I collagen, a biomarker of bone resorption, suggesting that iron overload enhances bone loss by activating osteoclasts [154]. These findings underscore the importance of iron in bone homeostasis, especially in osteoclasts.

### Targeting the lysosome–iron–mitochondria axis to modulate osteoclast activity

To mitigate bone loss in osteoclasts, the lysosome–iron–mitochondria axis could be the potential target. However, whether this axis should be considered as a whole or a separate target remains a question, even if it represents a tightly interconnected network in cellular processes, especially osteoclast-mediating bone resorption.

Firstly, lysosomal dysfunction can affect iron uptake, thereby leading to mitochondrial dysfunction, as mentioned above. Lysosomes, thus, can be the primary target for regulating osteoclast activity. A large body of evidence has demonstrated that it is possible to regulate osteoclast behavior by manipulating lysosomes [5,155–159]. For example, proper trafficking of lysosomes to the ruffled border is crucial for bone resorption. Proteins, such as Rab7 and RUFY4, are involved in regulating the movement of lysosomes along the cell membrane, and manipulating these proteins can regulate osteoclast bone resorption [157,159]. Meanwhile, osteopetrosis, a skeletal disorder with excessive bone mass caused by lysosomal disorders, can also provide side-proof for this concept [155,160]. Mutations in genes involved in lysosomal biogenesis or function can disrupt osteoclast resorptive activity, leading to too much bone mass. For instance, a mutation in CIC-7, which is involved in lysosomal acidification, has been linked to osteopetrosis [161]. Studies on this provide novel insights into the strategies that manipulate lysosomal behavior to regulate bone resorption.

Secondly, targeting iron availability is also an effective strategy to regulate osteoclasts and bone remodeling. Both iron deficiency and iron overload can disrupt the osteoclast activity. Growing evidence suggests that iron chelators can treat osteoporosis caused by iron overload by inhibiting osteoclasts [162,163]. Iron chelation therapy can also prevent osteopenia and osteoporosis in patients with thalassemia, estrogen deficiency, ionizing radiation, mechanical unloading, and Alzheimer’s disease [118]. It is worth noting that reduced iron uptake by TfR1 knockout [13] leads to osteoclast dysfunction similar to that of osteoclasts from osteopetrosis, indicating a missing link between lysosomes and iron in these dysfunctional osteoclasts. Exploring this missing link may help reveal the novel underlying mechanism of iron in the lysosome–iron–mitochondria axis, thereby improving our understanding of osteoclast biology.

Thirdly, manipulating mitochondria can also regulate osteoclasts. Deleting the Ndufs4 subunit of mitochondrial complex I can cause osteopetrosis [51]. Mitochondrial dysfunction, such as PGC1β deficiency [39,55], leads to similar osteoclast phenotypes in osteopetrosis patients, characterized by impaired osteoclast resorptive function but normal osteoclast formation. Similarly, osteoclasts deficient in TfR1 also display disrupted mitochondrial function and cytoskeletal organization, despite normal osteoclast differentiation and formation [13]. It is worth noting that these findings are similar to those observed in osteoclasts generated from lysosomal disorder diseases, which also exhibit impaired bone resorption with a lesser effect on osteoclast formation, suggesting a possible link between lysosomes, iron, and mitochondria within osteoclast-mediating bone resorption rather than osteoclast formation.

Despite the efficacy of targeting lysosomes, iron, or mitochondria individually, it is essential to recognize that these components are functionally interconnected; alterations in one often affect the other two. Lysosomes process and release iron for mitochondrial utilization, supporting energy production and ROS generation, which are necessary for osteoclast function. Therefore, it is meaningful to consider the lysosome–iron–mitochondria axis as an integrated regulatory network in osteoclasts. Disruption of this axis can impair osteoclast differentiation, resorptive activity, and survival, ultimately contributing to bone metabolic disorders. Understanding the dynamic crosstalk among these organelles offers new insights into osteoclast biology and presents a promising framework for therapeutic interventions targeting bone loss in diseases such as osteoporosis.

### Therapeutic application of targeting lysosome–iron–mitochondria axis in osteoclasts

In the context of osteoporosis, although it may be crucial to disrupt the lysosome–iron–mitochondria axis in osteoclasts to mitigate bone resorption, understanding the factors that can restore the functionality of this axis is also important for clinical practice.

Firstly, lysosomal acidification is essential for subsequent iron uptake, trafficking, and utilization. Therefore, restoring low lysosomal pH should benefit any dysfunction of the lysosome–iron–mitochondria axis by enhancing iron uptake. It has been confirmed that re-acidification of lysosomes can restore autophagy and mitochondrial function to lean, healthy levels in non-alcoholic fatty liver disease [164]. In addition, select members of the β2-adrenergic receptor (β2-AR) agonist family can also normalize elevated pH levels in PSEN1-deficient cells [165]. Notably, β2-AR agonists, such as isoproterenol, directly influence osteoclasts, promoting their maturation and increasing their bone-resorbing activity [166,167]. However, reagents that can normalize the lysosomal pH and restore the impaired lysosome–iron–mitochondria axis remain largely unexplored. On the other hand, whether or not increasing lysosomal biogenesis can also restore parts of lysosomal function remains unknown. However, in lysosomal disorders, there is an increasing number of lysosomes that may be a compensatory mechanism for the impaired lysosome function, but this increase is insufficient to restore normal lysosomal function.

Secondly, iron supplementation, especially ferric iron, has been shown to promote osteoclast differentiation and bone resorption [168]. However, we observed the opposite effects of iron supplementation, including FAC, on osteoclast differentiation (unpublished data). For the lysosome–iron–mitochondria axis, a recent study found that iron can restore cellular proliferation in the presence of lysosomal dysfunction but does not restore lysosomal pH-related catabolic and signaling functions [34]. However, iron supplementation can improve mitochondrial respiration, reduce mitochondrial damage, and enhance the cell’s ability to respond to stress [135].

Finally, enhanced mitochondrial function can also promote osteoclast differentiation and activity. Mitochondrial enhancers, such as the SIRT–JIP4 interaction, have also been shown to facilitate osteoclastogenesis [169]. Additionally, ROS, primarily generated by mitochondria, plays a key role in promoting osteoclast differentiation and function. This suggests that certain compounds capable of increasing intracellular ROS levels may influence osteoclast activity through mitochondrial pathways. Interestingly, mitochondria can, in turn, regulate lysosomal function and iron homeostasis, even though they are the endpoint of the lysosome–iron–mitochondria axis, as mentioned above. A recent study highlights the importance of the mitochondrial respiratory chain in CTSK production [8], suggesting that mitochondria may directly influence lysosomal activity. Supporting evidence from inflammatory T cell differentiation further reinforces the concept of mitochondria-mediated modulation of lysosomal function [170]. However, to date, no mitochondrial-targeted compounds have been reported to enhance osteoclast activity by restoring or boosting lysosomal function.

However, the therapeutic implications of modulating this axis may differ depending on the pathological context. In conditions characterized by excessive osteoclast activity, such as osteoporosis, the disruption of the lysosome–iron–mitochondria axis is desirable, while restoration should be avoided, as it may further promote bone resorption and compromise bone integrity. Conversely, in scenarios where osteoclast activity and function are impaired due to dysfunctional organelle communication or energy metabolism, like in certain forms of osteopetrosis, restoring or fine-tuning the lysosome–iron–mitochondria axis could be beneficial to normalize osteoclast function, improve bone modeling dynamics, and avoid pathological bone accumulation. Therefore, therapeutic modulation of the lysosome–iron–mitochondria axis must be context-specific and tightly controlled, potentially involving temporal or cell type-specific delivery strategies.

## Conclusion

The intricate interplay between mitochondria, iron, and lysosomes plays a crucial role in osteoclasts’ formation and function, directly influencing bone resorption and metabolism. Lysosomes are responsible for iron uptake, storage, and trafficking. Mitochondria are primary sites for iron utilization for energy production. Disruption of the lysosome–iron–mitochondria axis affects the functionality of lysosomes, iron metabolism, and mitochondria, ultimately impacting osteoclast-mediated bone resorption. Precision targeting of the lysosome–iron–mitochondria axis holds great promise as a therapeutic strategy for osteoporosis and osteoclast-related diseases.
